# Clinical Management of Patients with Heart Failure—2nd Edition

**DOI:** 10.3390/jcm14228122

**Published:** 2025-11-17

**Authors:** Cristina Tudoran

**Affiliations:** 1 Department VII, Internal Medicine II, Discipline of Cardiology, “Victor Babes” University of Medicine and Pharmacy Timisoara, Timisoara, E. Murgu Square, Nr. 2, 300041 Timisoara, Romania; tudoran.cristina@umft.ro; 2 Center of Molecular Research in Nephrology and Vascular Disease, “Victor Babes” University of Medicine and Pharmacy Timisoara, E. Murgu Square, Nr. 2, 300041 Timisoara, Romania; 3 County Emergency Hospital “Pius Brinzeu”, L. Rebreanu, Nr. 156, 300723 Timisoara, Romania

## Introduction

Heart failure (HF) is a complex pathology, being a consequence of multiple and intricated pathophysiological mechanisms that seldom occur on their own in the absence of other pathologies In the majority of cases, HF represents the outcome of other associated pathologies (coronary artery disease, systemic hypertension, diabetes mellitus, liver disease, chronic kidney disease, neurologic disorders) that coexist with HF, creating a vicious circle in which each disease is aggravated by the others [1,2,3,4,5]. To properly address the symptoms and outcomes of patients with HF, this Special Issue emphasizes HF′s risk factors and associated pathologies, but also the optimizations of specific HF therapies for individual patient categories.

Diabetes mellitus (DM) is an important risk factor for cardiovascular disease (CVD) and, ultimately, for the development of HF. Adopting a healthier, low-sugar diet can reduce the individual risk of developing DM. A Hungarian study demonstrated that artificial sweeteners were associated with increased CVD prevalence in patients with hypertension, coronary artery disease, and stroke [6].

As mentioned above, HF may be induced or aggravated by pre-existing chronic or acute pathologies [7]. As stated by Lupu et al., liver cirrhosis may determine the onset and evolution of cirrhotic cardiomyopathy (CCM), worsening the outcome of these patients and influencing therapeutic decisions [8]. The study focuses on the complex mechanisms that influence the development of CCM, aiming to elaborate targeted therapeutic strategies for individuals suffering from CCM. Two other authors highlighted the burden that infections place on the outcomes of patients with HF [9,10], an aspect frequently debated in the medical literature [11]. Healthcare-associated infections, especially in patients hospitalized in intensive care units, may lead to sepsis, which then determines the development of episodes of acute HF, challenging the treating physicians to timely identify any signs of decompensated HF and implement guideline-directed medical therapy as soon as possible to prevent further complications and poor evolution of the patient.

The outcome of HF with reduced ejection fraction has consistently improved in recent decades due to the implementation of the European Society of Cardiology (ESC) guidelines [12,13] regarding the therapy of HF with disease-modifying drugs such as angiotensin-converting enzyme inhibitors (ACE-Is) or angiotensin receptor–neprilysin inhibitors (ARNIs), beta-blockers, and mineralocorticoid receptor antagonists (MRAs). These drugs have been shown to improve survival, reduce the risk of HF hospitalizations, and reduce symptoms in HF patients [14]. Significant additional benefit has been obtained after the introduction of the of sodium–glucose co-transporter 2 (SGLT2) inhibitors (Class IA recommendation) [12,13]. Therefore, the monitoring of the efficiency of these therapies is crucial, and new biomarkers are required [15]. Besides NT-pro BNP, monitoring sST2 might offer new opportunities for therapy guidance and disease management [16].

Chronic patient-centered disease self-management programs aiming to educate patients on HF management, their ability to self-monitor symptoms, and goal-setting have ameliorated outcomes, especially in patients with HF with reduced ejection fraction. Due to inconsistent reporting in the medical literature regarding the results and components from studies on self-management, the exact benefit of these approaches has yet to be determined [17].

In recent years, iron deficiency has been considered a comorbidity in HF patients. Iron deficiency has been proven to reduce the parameters of left ventricular systolic function (global longitudinal strain (GLS) and myocardial work), especially in patients with reduced ejection fraction. Right ventricular function, especially RV-free wall strain and RV coupling, also declines. Fortunately, these changes seem to reverse after iron therapy, especially after intravenous repletion with ferric carboxymaltose [18]. In patients with HF, CoQ10 supplementation, as an adjuvant therapy, may ameliorate cardiac function, functional capacity, quality of life, and cardiac stress by improving GLS, NT-pro BNP levels, blood pressure, and the distance achieved in a 6 min walk test [19].

One of the main goals of the management of HF is to lower the rate of hospital readmissions for acute/decompensated HF [20]. To reduce the rate of readmissions due to repeated episodes of acute HF, a patient-centered, dynamic, multidisciplinary approach that is adapted to the different risk profiles of each patient is required. Future therapeutic strategies should focus on non-pharmacological options (dietary modifications, exercise, cardiac rehabilitation, and structured follow-up programs) [21,22].

The roles of the different categories of cholesterol have long been debated among scholars. Although high-density lipoprotein (HDL) has cardioprotective and anti-atherosclerotic properties, its quality, rather than its concentration, may be more important. On the other hand, small dense low-density lipoproteins (sdLDLs) are deemed to be highly atherogenic, considering their enhanced arterial penetration, oxidative susceptibility, and prolonged plasma residence time. Current lipid management strategies recommend aggressively lowering LDL-C and apoB-containing lipoproteins using statins, ezetimibe, PCSK9 inhibitors, fibrates, and structured lifestyle changes to increase HDL levels and functionality in patients with CVD [23].

Emphasis should be placed on associating the optimal medical treatments with intracardiac devices to enhance cardiac function in patients with HF [24]. Between 20 and 40% of patients with cardiac resynchronization therapy (CRT) are non-responders. Overall patient profile plays a significant role, along with HF severity and the burden of comorbidities [25]. This data highlights the need to optimize the recommended CRT protocols. ARNI therapy in combination with CRT may improve left ventricular ejection fraction (LVEF), NYHA functional class, and ventricular remodeling, particularly in CRT non-responders. Some studies also report a potential reduction in ventricular arrhythmias and the need for implantable cardioverter-defibrillator (ICD) interventions [26].

In patients suspected to be non-responsive to the biventricular pacing mode of traditional CRT, left-ventricle-only fusion pacing (LV-only fCRTp) may optimize the patient′s response and offer long-term therapeutic benefits. Combining left ventricular epicardial pacing with the intrinsic ventricular activation wavefront (LV-only fCRTp) could offer an alternative to traditional biventricular pacing. Careful selections and proper optimizations, such as fusion strategies, are necessary to fully achieve the potential of LV-only fCRTp. The most important criterion for LV-only fCRTp is the preservation of AV conduction, a fundamental condition for achieving and maintaining an effective electrical fusion between intrinsic RV activation and LV pacing. The etiology of CVD and the assessment of arrhythmic risk in patients with HF and preserved ejection fraction play a significant role in determining the type of CRT that is necessary [25].

## Data Availability

All of the data are presented in the manuscript.
